# Attitudes and Behaviors That Impact Skin Cancer Risk among Men

**DOI:** 10.3390/ijerph18199989

**Published:** 2021-09-23

**Authors:** Gabrielle J. Adams, Elianna K. Goldstein, Beth G. Goldstein, Kristen L. Jarman, Adam O. Goldstein

**Affiliations:** 1 College of Arts and Sciences, University of North Carolina at Chapel Hill, Chapel Hill, NC 27599, USA; gabriellea@centraldermcenter.com; 2 Skinvest, Inc., Chapel Hill, NC 27517, USA; elianna@getmr.com; 3 Department of Dermatology, School of Medicine, University of North Carolina at Chapel Hill, Chapel Hill, NC 27599, USA; beth@getmr.com; 4 Department of Family Medicine, School of Medicine, University of North Carolina at Chapel Hill, Chapel Hill, NC 27599, USA; jkristen@email.unc.edu

**Keywords:** sunscreen, men, prevention, behavior, skin cancer

## Abstract

Despite substantially higher skin cancer risks, little research has investigated men’s attitudes about skin cancer and how those attitudes relate to their risks of developing skin cancer. This study aims to close the gap in research, regarding men’s perceptions and behaviors about skin cancer, sun exposure, and tanning. This study utilized a cross-sectional survey of 705 men recruited from Amazon Mechanical Turk (MTurk), reporting attitudes and behaviors towards sun exposure, tanning, and sun protection. While the majority of men reported large daily outdoor activities, that their skin frequently burns with sun exposure, and riskier perceptions of tanning, only a minority reported daily use of sunscreen or most other sun protective behaviors. More sun protection methods were associated with more frequent use of sunscreen and less positive tanning perceptions. Men consistently engaged in high-risk behaviors for developing skin cancer, but they did not engage highly in protective behaviors to mitigate their risk. The findings can help improve clinical and public health interventions to lower men’s risk of skin cancer with strong messages about sunscreen use and sun protective methods.

## 1. Introduction

Skin cancer is the most common cancer in the world, with more than 5 million cases diagnosed in the United States annually [1,2]. The majority of skin cancers are basal cell or squamous cell carcinomas that cause substantial morbidity and are associated with increased mortality from other cancer types [3]. Basal cell and squamous cell carcinomas have increased by 145% and 263%, respectively, over the last three decades [4]. In addition, almost 200,000 new cases of melanoma skin cancers occur annually [5].

Despite high and increasing cases of skin cancer, patients are typically not well-informed of their risk factors, such as a family history of skin cancer, exposure to excess UV radiation, lower Fitzpatrick skin scores, frequent sun exposure, or available prevention strategies [6]. Research clearly shows the carcinogenic potential of UV light, and the popularization of purposeful UV exposure with tanning has significantly increased patient risk for developing both melanoma and non-melanoma skin cancers [7]. Some health professionals have insufficient knowledge of concomitant use of sunscreen, ultraviolet (UV)-light exposure, and skin cancer risk [8].

By 2040, melanoma will be the second most common cancer overall and the most common cancer among men (excluding non-melanoma skin cancer) [9]. Men have disproportionately higher rates of skin cancer and typically experience worse outcomes following a skin cancer diagnosis [10]. Reasons behind this difference are often behavioral, as men tend to apply sunscreen less frequently than women and use less sun protective clothing [11]. Some of these behaviors may result from a lack of knowledge of the risks [12], but even after a melanoma diagnosis, men are less likely to adopt skin-protective behaviors [13]. 

Despite higher risks, little research has investigated men’s attitudes about skin cancer and how those attitudes relate to their risks of developing skin cancer. Our research investigates men’s reports on their sun exposure and perception of their skin cancer risks, including tanning, and their skin cancer prevention behaviors. This research may help in devising improved strategies to lower men’s rates of skin cancer. 

## 2. Materials and Methods

Participants for this study were recruited, using Amazon Mechanical Turk (MTurk), a validated data collection and research marketplace [14]. Subjects were eligible for the study if they were MTurk members, were between 20 and 70 years old, had a yearly income of over 40,000, currently lived in the United States, and had graduated from high school. Participants completed a 15-minute questionnaire, regarding their daily exposure to the sun, skin type, sun protection behaviors, and attitudes toward tanning [15]. Participants were eligible to receive a USD 3 Amazon credit upon completion of the survey. Due to this study focusing on men, all participants who do not identify as men were removed from this analysis. Additional details for how the sample was collected are described in a previous paper [11]. A full list of measures for this study is included in a previous paper [11].

Participant’s sun exposure was assessed, using routine occupational and non-occupational outdoor activities. To assess non-occupational outdoor activity, participants selected outdoor activities that they regularly spent time doing, including the following: going to the beach; pool/swimming; water sports (boating, sailing, windsurfing, fishing); walking; running; watching a sport; cycling/biking; and gardening. Participants were also asked what sports they spent time doing outside: they were asked about playing golf, racket sports (tennis, badminton, and pickleball), soccer, football, baseball or softball, or other. Many participants indicated in the other column that they play basketball outside, so basketball was also added to this category based on responses to the ‘other’. Participants were also asked if they spend 4 hours or more per day outside for work. To create a score for sun exposure, each activity that a participant reported to participate in was given a 1, and all activities were summed. Participants that reported spending 4 or more hours per day outside for work had an additional 4 points added to their sun exposure score.

Skin type was assessed by questions from the well-established Fitzpatrick scale [16] about how their skin responds to the sun and if their skin tans. A skin type score was created by assigning each question a range of 0 to 4, where 0 represents a person who never burns and always tans, and a 4 is a person who always burns and never tans; the two skin type questions were then summed to create a score ranging from 0 to 8.

Sun protective behavior was assessed by asking participants modified questions on how often they use sun protective behaviors [15] when they spend more than one hour outside on a warm sunny day, using a 5-point Likert scale. Sun protective behaviors included the following: stay in the shade; wear a baseball cap or sun visor; wear a wide-brimmed hat; wear a long-sleeved shirt; wear long pants or other clothing that covers the ankles; use sunscreen; or wear sunglasses. To calculate the sun protective behavior mean, each response option was assigned a score such that an answer of always was assigned 4 points, and never was assigned 0 points, and the mean of all 7 types of sun protection responses was calculated. Participants, using multiple methods of sun protection frequently would score higher, and participants using fewer sun protection methods, or using sun protection methods less frequently would score lower because they are doing less to mitigate their risk of sun damage. Additionally, the use of sunscreen on the face was assessed by asking how often participants use sunscreen on their face, and dichotomizing to participants that use it daily or weekly, and participants that use less than weekly.

Attitudes toward tanning was assessed, using a series of validated questions [15] in which participants were asked to what extent they agreed with a series of statements regarding tanning, using a 5-point Likert scale (1 = strongly disagree to 5 = strongly agree). Risky tanning perceptions include the following: most of my friends think a tan is a good thing; a tan makes me feel better about myself; I feel more healthy with a tan; tanning makes me look more attractive; tanning makes me look younger; and tanning makes my skin look more even. Participants were also asked to what extent they agreed with the statement: tanning makes me look older. A score was created where responses to all the risky tanning perception questions were summed, and perceptions that tanning made the participant look older was subtracted. A higher score indicates riskier perceptions around tanning.

All statistical analyses were conducted, using SAS 9.4 (SAS, Cary, NC, USA). Descriptive statistics of responses across sun exposure, skin type, sun protection behaviors, facial sunscreen use, and perceptions of tanning were calculated. A logistic regression model was estimated with the use of facial sunscreen daily or weekly as the outcome, predicted by sun exposure, sun protection, tanning perceptions, skin type and demographic factors. Linear models were estimated with outcomes of sun exposure, sun protection behaviors, and perceptions of tanning with other risk factors and demographic information included, using proc glm9.

## 3. Results

Respondents included a total of 705 men of whom 69% were white, 17% black, and 15% other. Additionally, 78% of our sample identified as straight or heterosexual, and 16% of our sample identified as Hispanic. Most participants were between 20 and 39 (60%), and 59% reported an income between USD 50,000 and 100,000 annually. In response to sun exposure, 74.4% of men reported that their skin type either always, often or moderately burns, blisters or peels (Table 1). A minority of men reported that their skin type rarely (21.7%) or never (3.8%) burns in response to the sun. Only 17.1% of men reported daily use of sunscreen, 38.6% reported weekly use, 16.1% reported monthly use, and 28.3% reported using sunscreen infrequently. 

Participants’ daily sun exposure is shown in Table 2. Over half of participants spent significant time outside at the beach, in a pool, walking and socializing with friends. One third (32.4%) reported spending 4 or more hours a day outdoors at work. 

Men’s perceptions of tanning are shown in Table 3. The majority of men reported riskier perceptions of tanning for most tanning perceptions. The discrepancy was largest for the statement that “most of my friends think a tan is a good thing”, where four times more men agreed or strongly agreed with the statement than disagreed or strongly disagreed. 

Men’s reports on sun protective behaviors are shown in Table 4. With the exception of wearing sunglasses, most men did not use any other sun avoidance behaviors always or most of the time. 

In regression models (Table 5), significant associations existed between sun exposure, tanning perceptions, sun protection, and sunscreen. Higher sun exposure was associated with more positive perceptions of tanning, more frequent use of sunscreen and sun protection, and a skin type that was more likely to burn. Higher sun exposure was also associated with those who identified as Black or African American as well as those who identified as Hispanic or Latino/a. More positive perceptions of tanning were associated with less frequent use of sun protection and increased sun exposure, as well as skin type less likely to burn. Positive tanning perceptions were also higher among people who identified as Hispanic or Latino/a.

More sun protection methods were associated with more frequent use of sunscreen, less positive tanning perceptions, and increased sun exposure. Frequency of sun protection methods was higher among people who identified as Hispanic or Latino/a and among people ages 50 to 70. Higher use of sunscreen was associated more frequent use of sun protection but also higher sun exposure. Sexual orientation was not found to be a predictor of any outcome investigated.

## 4. Discussion

Extensive research exists on risk factors associated with the development of skin cancer, including higher ultraviolet light exposure [17], skin types with an increased tendency to burn [18], and the use of fewer sun protection behaviors [19]. Higher risks of serious skin cancer among men are related, in part, to their lower rates of sunscreen and sun protection methods [19]. To date though, little research exists on men’s perception of their risk and the behaviors that they implement because of these perceptions. These perceptions are critical, as most men do not use sunscreen on a regular basis, despite their acknowledgement that a primary factor that motivates many of them to wear sunscreen is to reduce their risk of developing skin cancer [11]. 

Our research shows that men in our sample spent extensive time outdoors, most on a daily basis. They also endorsed more positive, or riskier, perceptions of tanning, and the majority reported that their skin type frequently burns in response to sun exposure. Even with elevated risks, few reported regular use of most sun avoidance behaviors, and positive tanning perceptions were associated with increased sun exposure and less frequent sun protection. These results have important implications for efforts to increase men’s sun protection behaviors and reduce their skin cancer risk.

Strong associations between increased sun protection methods and less positive tanning perceptions suggest specific areas that clinicians should focus when counseling their male patients. Changing men’s perceptions of tanning may be difficult, but unequivocal recommendations by clinicians make a difference. For instance, sun protection counseling by a dermatologist increases patient’s daily or weekly sunscreen use [20,21]. Counseling about sun protection and tanning, combined with provision of free sunscreen, is likely effective in populations typically more resistant to counseling, such as older men [21]. Finally, research shows that riskier perceptions towards tanning are more amenable when such counseling is given verbally [22]. By knowing these areas of emphasis, clinicians can emulate other very successful clinical interventions, such as counseling patients to quit tobacco or alcohol [23].

Little research exists on broad-based efforts to provide sun protective behavioral counseling in dermatology or primary care offices, even for patients with significant risk factors for skin cancer [24]. Given that melanoma will likely be the number one cancer among men within 20 years [8], incorporating counseling about broader ways to reduce skin cancer risk should be incorporated into the annual men’s wellness exam, especially for young adults, where counseling is especially effective [25]. Current guidelines regarding sun protection counseling note that sun protective counseling in adults is effective and should be targeted towards high-risk individuals [20]. Interventions facilitated by primary care physicians and pediatricians can also increase sun protective counseling being given at visits [26]. As previously mentioned, while incidence rates of melanoma are expected to rise within the next 20 years, death rates are steadily decreasing [8]. This distinction, however, does not negate the prevalence or importance of melanoma risk nor make counseling towards its prevention inconsequential. 

Educating physicians on perceptions and behaviors that predict increased risk for future skin cancers would also allow physicians to target specific high-risk patients with strong messaging on sun protective behaviors. For example, in our study, Hispanic participants demonstrated higher sun exposure and more positive perceptions of tanning but also more sun protective behaviors. This finding suggests that this population may benefit from more targeted counseling about increasing sun protection rather than on changing tanning risk perceptions.

Community-based interventions may also change unhealthy perceptions of tanning or more frequent use of sunscreen. For instance, a community-based intervention designed for increasing sun protection behaviors at golf courses, an outdoor activity used more frequently by men, showed positive self-reported behavioral impacts [27]. However, most community-based interventions are targeted towards adolescents [28], including a study with wearable UV exposure monitoring devices and free sunscreen that led to more frequent use of sunscreen and sunglasses [29]. It is unknown whether these interventions would appeal to or have the same outcomes among adult men.

Widespread sun protection communication campaigns using multichannel methods may complement clinical and community-based messaging. Specifically, these campaigns appear effective at increasing some sun protective behaviors, such as applying sunscreen and lip balm [30]. Currently designed public health campaigns have limited reach, however, limiting patients’ abilities to recall seeing messages, and most have been targeted to adolescents, not high-risk adults [31]. Campaigns appear less effective at increasing participant’s feelings of self-efficacy, regarding developing skin cancer or changing riskier attitudes towards sun exposure [25]. Newer forms of communication that involve social media and smart phones may offer more targeted approaches to reaching high-risk populations [32,33,34,35]. While public policy efforts, such as placing age limit restrictions of tanning bed use have shown to be effective in limiting tanning bed use, few policy interventions to date have focused on reducing skin cancer risks among men [36]. 

Future research should assess specific clinical, community-based, and media interventions that may increase men’s utilization of and compliance with sunscreen use, as well as increased sun-protective behaviors outside of sunscreen use. Longitudinal studies are particularly needed to show how changing men’s behaviors and perceptions may reduce future skin cancer rates. 

Limitations to this research exist. While cross-sectional correlations were found, due to the nature of this study, it is unclear based on the study design whether sun exposure, sun protection behaviors, or tanning risk perceptions have a causal relationship with each other, or which way any causal relationship occurs. As our study captured more men with lighter skin types due to their increased risk of skin cancer, our sample could be less generalizable to the perceptions of men with darker complexions [37]. Our study measures of outdoor sun exposure may be higher than usual, as the majority of data were collected during summer months, when incidences of sun exposure are higher. Our study did not capture the specifics of men’s tanning behaviors, such as if they use sunless tanning, tanning beds, or UV radiation from the sun [38]. Finally, we did not assess the haircuts or grooming preferences of our sample, such as if they had a shaved head or beard, which may impact their risk for developing skin cancer on their head, neck, or face [39]. It is also possible that men used products that incidentally contained sunscreen.

## 5. Conclusions

This study adds to a growing body of literature that focuses on perceptions and behaviors that predict skin cancer risk among men. This research is needed, as men are diagnosed with more cases of non-melanoma and melanoma skin cancer each year, compared to women [40]. Concrete and specific behaviors exist that male patients can implement to drastically reduce their risk for skin cancer [41]. The misalignment among many men between risk, perception of risk, and behaviors to reduce risk should be future targets for increased clinical and public health interventions. 

## Figures and Tables

**Table 1:** Participants’ skin type assessment (*n* = 705).

How does your skin respond to the sun?	Percent	*N*
Always burns, blisters, and peels	5.5	39
Often burns, blisters, and peels	18.3	129
Burns moderately	50.6	357
Burns rarely, if at all	21.7	153
Never burns	3.8	27
**Does your skin tan?**	**Percent**	***N***
Never, I always burn	5.4	38
Seldom	14.5	102
Sometimes	33.8	238
Often	31.4	221
Always	15.0	106

**Table 2:** Participants’ regular outdoor sun exposure activities (*n* = 705).

Activity	Percent	*N*
Walking	76	536
Going to the beach	50.9	359
Going to the pool/swimming	50.5	356
Running	43	303
Watching a sport (baseball, soccer, football, etc.)	37	261
Gardening	34.6	244
Work outside 4 or more hours per day	32.2	227
Cycling/Biking	31.5	222
Golf	25.7	181
Tennis/Badminton/Pickleball	25.1	177
Football	24	169
Baseball or Softball	22.4	158
Boating, sailing, windsurfing, surfing, fishing	22	155
Soccer	18.7	132
Basketball	6.5	46

**Table 3:** Men’s perceptions toward tanning (*n* = 705).

	Strongly Disagree, Somewhat Disagree		Neither Agree Nor Disagree		Somewhat Agree, Strongly Agree	
Question	Percent	*N*	Percent	*N*	Percent	*N*
Most of my friends think a tan is a good thing	15.4%	105	22.9%	156	61.7%	420
A tan makes me feel better about myself	28.8%	196	24.7%	168	46.6%	317
I feel more healthy with a tan	32.7%	223	25.0%	170	42.3%	288
Tanning makes me look more attractive	22.3%	152	25.3%	172	52.4%	357
Tanning makes me look younger	35.5%	242	30.7%	209	33.8%	230
Tanning makes my skin look more even	25.6%	174	30.7%	209	43.8%	298
Tanning makes me look older	38.2%	260	30.4%	207	31.4%	214

**Table 4:** Participants’ sun protective behavior when in the sun for > an hour (*n* = 705).

	Always, Most of the Time		Sometimes		Rarely, Never	
	Percent	*N*	Percent	*N*	Percent	*N*
Stay in the shade?	28.3%	196	51.2%	354	20.4%	141
Wear a baseball cap or a sun visor?	40.2%	278	27.0%	187	32.1%	222
Wear a hat that shades your face, ears AND neck such as a hat with a wide brim all around?	13.9%	96	19.9%	138	65.5%	453
Wear a long-sleeved shirt?	13.0%	90	21.7%	150	64.3%	445
Wear long pants or other clothing that reaches your ankles?	25.3%	175	23.1%	160	50.6%	350
Use sunscreen?	48.0%	332	34.8%	241	16.6%	115
Wear sunglasses?	53.5%	370	21.2%	147	24.9%	172

**Table 5:** Predictors of use of facial sunscreen, sun exposure, sun protection behaviors, and perceptions of tanning.

Use of Facial Sunscreen Daily or Weekly (Logistic Regression)					
Variable		OR	95% CI		*p*-Value
Sun Exposure		1.10	1.04	1.16	0.0005
Sun Protection		2.60	1.97	3.44	<0.0001
Race	Black or African American	0.49	0.30	0.80	0.004
	Other	0.82	0.51	1.31	0.40
	White (ref)	-	-	-	-
Hispanic or Latino (ref–not Hispanic or Latino)		0.55	0.34	0.89	0.02
**Sun Exposure–Higher is More Activities Outside (Linear Regression)**					
**Variable**		**Estimate**	**95% CI**		***p*-Value**
Sun Protection		0.64	0.26	1.02	0.001
Tanning Perceptions		0.08	0.04	0.12	0.0002
Use of Facial Sunscreen Daily or Weekly (ref–uses facial sunscreen less than weekly)		0.89	0.40	1.38	0.0004
Skin Type–higher is higher likelihood of burning		0.16	0.01	0.32	0.03
Race	Black or African American	1.35	0.66	2.03	0.0001
	Other	0.34	−0.34	1.03	0.32
	White (ref)	-	-	-	-
Hispanic or Latino (ref - not Hispanic or Latino)		0.80	0.10	1.50	0.03
**Sun Protection–Higher is More Frequent Use of Sun Protection Methods (Linear Regression)**					
**Variable**		**Estimate**	**95% CI**		***p*-Value**
Sun Exposure		0.02	0.01	0.04	0.001
Sun Protection		-	-	-	-
Tanning Perceptions		−0.01	−0.02	0.00	0.0172
Use of Facial Sunscreen Daily or Weekly (ref–uses facial sunscreen less than weekly)		0.34	0.25	0.43	<0.0001
Hispanic or Latino (ref–not Hispanic or Latino)		0.15	0.01	0.29	0.03
Age Group	20–29 (ref)	-	-	-	-
	30–39	−0.02	−0.14	0.10	0.75
	40–49	0.03	−0.10	0.16	0.62
	50–59	0.21	0.04	0.38	0.0154
	60–70	0.22	0.01	0.42	0.0425
**Tan Perceptions–Higher is More Positive Associations with Tanning (Linear Regression)**					
**Variable**		**Estimate**	**95% CI**		***p*-Value**
Sun Exposure		0.26	0.13	0.40	0.0002
Sun Protection		−0.85	−1.56	−0.15	0.0172
Skin Type–higher is higher likelihood of burning		−0.56	−0.84	−0.29	<0.0001
Race	Black or African American	−0.02	−1.29	1.26	0.98
	Other	−1.49	−2.74	−0.24	0.02
	White (ref)	-	-	-	-
Hispanic or Latino (ref–not Hispanic or Latino)		1.33	0.04	2.62	0.04

## Data Availability

All experimental data to support the findings of this study are available upon request from the corresponding author.
